# Analysis of glutathione-S-transferases from larvae of *Galleria mellonella* (Lepidoptera, Pyralidae) with potential alkaloid detoxification function

**DOI:** 10.3389/fphys.2022.989006

**Published:** 2022-09-06

**Authors:** Herbert Venthur, Paula Lizana, Loreto Manosalva, Valentina Rojas, Ricardo Godoy, Adonis Rocha, Iván Aguilera, Rubén Palma-Millanao, Victor Fajardo, Andrés Quiroz, Ana Mutis

**Affiliations:** ^1^ Departamento de Ciencias Químicas y Recursos Naturales, Facultad de Ingeniería y Ciencias, Universidad de La Frontera, Temuco, Chile; ^2^ Centro de Investigación Biotecnológica Aplicada al Medio Ambiente, CIBAMA, Universidad de La Frontera, Temuco, Chile; ^3^ Programa de Doctorado en Ciencias de Recursos Naturales, Universidad de La Frontera, Temuco, Chile; ^4^ Facultad de Ciencias, Universidad de Magallanes, Punta Arenas, Chile; ^5^ Carrera de Bioquímica, Departamento de Ciencias Químicas y Recursos Naturales, Universidad de La Frontera, Temuco, Chile; ^6^ Laboratorio de Ciencias de Insectos, Instituto de Investigaciones Agropecuarias, INIA, Vilcún, Chile

**Keywords:** pest control, enzymatic activity, *Galleria* mellonella, alkaloids, glutathione-S-transferase (GST)

## Abstract

The greater wax moth, *Galleria mellonella*, is a global pest for beehives, doing damage in the larval stage. Although a significant number of studies have reported on larvae and adults, to date no effective pest control has been implemented. In this study, we tested larval resistance to alkaloids from *Berberis microphylla*, and the objective was to identify enzymes that participate in alkaloid detoxification through enzymatic assays, bioinformatics analysis and qRT-PCR. Findings suggest glutathione-S-transferases (GSTs), from an increased metabolic mechanism, are responsible for alkaloid detoxification rather than cytochrome P450 (CYP), carboxylesterases (CarE). A bioinformatics analysis from transcriptome data revealed 22 GSTs present in both *G. mellonella* larvae and adults. The qRT-PCR experiments corroborated the presence of the 22 GSTs in larvae, where GST8 and GST20 stood out with the highest expression after berberine treatment. Structural information around GST8 and GST20 suggests that GST8 could bind berberine stronger than GST20. These findings represent an important advance in the study of detoxification enzymes in *G. mellonella*, expanding the role of delta-class GSTs towards alkaloids. Likewise, GST inhibition by alkaloid analogs is proposed in the framework of integrated pest management strategies.

## Introduction

Nowadays, honey bees are of global concern based not only on millions of tons of honey production threatened by intensive agriculture (e.g., systemic use of pesticides) and global warming effects (e.g., spread of pests) (Le Conte and Navajas, 2008; Godfray et al., 2015; Cornelissen et al., 2019), but also due to the pollination process, proven to positively impact fruit set (Garibaldi et al., 2013). Factors that have negatively affected the quality of colonies, bee health and honey production include insects, such as the greater wax moth *Galleria mellonella* (Lepidoptera: Pyralidae). This stands out as a worldwide distributed moth and as the most abundant beehive pest among insects, feeding on honey and wax at the larval stage (Nielsen and Brister, 1979).

Traditional methods for controlling *G. mellonella* populations have included the use of pesticides (e.g., pyrethroids), and the sterile male technique, where gamma rays are used to sterilize males of the moth with the subsequent inability to procreate (Jafari et al., 2010). Moreover, azaridachtin from alcoholic extracts of Neem-X^®^ (a biopesticide derived from the tree *Azaridachta indica*) has been tested with some promising antifeedant effects at the larval stage (Contreras, 2011). Likewise, a biological control method using *Bacillus thuringiensis* as well as practical methods like constant bee feeding, reduction of either super or unoccupied frames have been reported (Tsegaye et al., 2014; Gela et al., 2017). More recently, an aggregation pheromone (i.e., decanal) has been reported as a promising semiochemical to control *G. mellonella* larvae. However, these findings rely on olfactory mechanisms that have recently started to be revealed only for *G. mellonella* adults (Zhao et al., 2019; Aburto et al., 2020; Lizana et al., 2020). Overall, none of the previous methods have resulted in the efficient control of *G. mellonella*.

Alternative insect control methods have emerged due to the advance in understanding of their enzyme-based detoxification system. The resistance to insecticides that the peach potato aphid *Myzus persicae* has acquired due to, for instance, the overexpression of carboxylesterases, is a good starting point (Bass et al., 2014). In moths, particularly the codling moth *Cydia pomonella*, two resistance mechanisms have been proposed, namely target-site and metabolic mechanisms (Ju et al., 2021). Thus, common detoxification enzymes from an increased metabolism are carboxylesterases (CarE), cytochrome P450 (CYP) and glutathione-S-transferases (GSTs), while from target-site mutations are acetylcholinesterases (AChE). In particular, GSTs comprise a large family of enzymes that participates primarily in the detoxification of endogenous compounds and xenobiotics. For instance, the silkworm moth *Bombyx mori* GST, bmGSTu2, was reported to metabolize diazonin, an organophosphate insecticide (Yamamoto and Yamada, 2016). Similarly, a detoxification ability against organophosphate insecticides, chlorpyrifos and dichlorvos, has been reported for the *Helicoverpa armigera* GST, HaGST-8 (Labade et al., 2018). In addition to the insecticide-detoxifying role of GSTs, odorant-degrading functions have been related to these types of enzymes when located in antennae (Durand et al., 2018). Interestingly, *G. mellonella* has a particularly complex profile of enzymes due to its powerful digestive system at the larval stage. An interesting fact is its ability to degrade long-chain hydrocarbon compounds (e.g., polyethylene), where enzymes such as CYP, carboxylesterases, and lipases, are related (Kong et al., 2019). In relation to GSTs in *G. mellonella*, early research suggests the presence of multiple enzymes of this type in larvae (Baker et al., 1994). Their role in enhancing resistance and immunity against entomopathogenic fungi (Serebrov et al., 2006) and dietary nickel (Dubovskiy et al., 2011), respectively, has also been reported. However, a deeper knowledge around *G. mellonella* GSTs, such as number of enzymes, molecular characteristics, enzymatic activity and structure, is still lacking. In addition, it is worth noting that moth GST function is mainly related to insecticides and semiochemicals, with less focus on alkaloids that could function as control agents. These alkaloids have shown some interesting effects on some moth species at the larval stage. For instance, alkaloids antofine *N*-oxide, antofine and tylophorine decreased the pupation and emergence rate of *Spodoptera litura* larvae (Ge et al., 2015). Berberine, an isoquinoline alkaloid, showed potent antifeedant activity on larvae of the gypsy moth *Lymantria dispar* (Shields et al., 2008). Nevertheless, to date these types of chemicals have not been linked to detoxification processes (i.e., GSTs).

In this study, experiments were focused on the larval stage of *G. mellonella*, considered the most harmful stage for beehives. Here, we hypothesize one main GST to be responsible for detoxification in *G. mellonella* larvae, and this could be a target for future research into integrated pest management strategies. Therefore, the objective was to characterize *G. mellonella* GSTs activity at the functional, molecular and structural levels against selected alkaloids. First, we targeted four enzymes (i.e., AChE, CYP, CarE, and GST) using alkaloid extracts as the means to identify highly active ones. Then, bioinformatics methods for identifying GSTs were applied, followed by relative expression experiments through qRT-PCR. Finally, protein structure prediction was performed to clarify putative interactions between enzymes and alkaloids.

## Materials and methods

### Plant material and alkaloid extraction

Representative samples of roots of *Berberis microphylla* (syn. *Berberis heterophylla*) were collected during the flowering season at Comodoro Rivadavia in Argentinian Patagonia (45°56.7′ S; 67°35.6′W) in January 2012. A voucher specimen was deposited at the Herbario Regional Patagónico of the Facultad de Ciencias Naturales, Universidad Nacional de la Patagonia San Juan Bosco (UNPSJB), *Argentina* (Voucher N°7293). The plant material was vacuum-packed and stored at −80°C for further study. Extraction was carried out according to the methodology described by Cabezas et al. (2009) with some modifications. Oven-dried and powdered roots (300 g) of *B. microphylla* were sequentially extracted (24, 48 and 72 8) with methanol at room temperature. Methanolic extracts were vacuum evaporated at 40°C, and the residue was reconstituted with 200 ml 10% hydrogen chloride for 1 h under agitation (orbital shaker, MS-NOR, Taiwan) and left to stand for 12 h at 10°C and then filtered. The filtrate was washed with chloroform (5 ml × 100 ml). The aqueous phase was adjusted to pH 10 with ammonium hydroxide and extracted with chloroform (5 ml × 100 ml). The solvent was evaporated to obtain the extract containing alkaloids.

### HPLC ESI-MS/MS analysis

The chromatographic separation was carried out using a RP-C18 BioSuite column (2.1 mm × 150 mm, 3 μm), injecting 10 μL at 0.2 ml/min and 35°C. 0.01 g of standards, and sample extracts were dissolved in 10 ml of methanol and subjected to LC–MS/MS. The chromatographic separation was performed using a linear gradient solvent system consisting of 0.1% formic acid (A) and acetonitrile (B). The linear gradient was composed of 0–3 min 10% B, 3–35 min 10–70% B, 35–40 min 70% B, 40–50 min 70–10% B, then again under the initial conditions (10% B) for 10 min. Each standard was injected with an electro spray ionization (ESI) source into the mass spectrometer (LC-MS MS Shimadzu Prominence coupled to Applied Biosystems/MDS Sciex3200 Qtrap mass spectrometer, Massachusetts, United States). The ion source temperature was set to 400°C, and the capillary voltage was 5.5 kv. For alkaloid determination, data were collected as positive-ion spectra by means of enhanced mass scan (EMS) over a m/z 100–1,000 Da range at 1,000 Da/s and Enhanced Product Ion (EPI) over a m/z 50–1,000 Da range at 4,000 Da/s. The CUR gas was 20 psi, GS1 30 psi and GS2 60psi.

### G. mellonella larvae and exposure to alkaloids

Larvae of *G. mellonella* were kept under rearing conditions in the Laboratorio de Química Ecológica, Universidad de La Frontera, Chile, from September 2019 to December 2021, using plastic boxes covered by metallic mesh in which a diet consisting of cereal, honey, vitamins, glycerin and distilled water was provided following the instructions reported by Zamorano (2009). Likewise, larvae were kept in a growth chamber at 25°C with a 14 h scotophase. Preliminary bioassays were performed with third instar larvae exposed to alkaloid extracts as well as palmatine and berberine (20 ppm each). Thus, larvae were kept in plastic boxes with the afore mentioned diet plus alkaloids and water as negative control. Effects such as mortality, pupation and diet ingestion were recorded for 7 days.

### Treatments for enzymatic assays

To prepare the test diet, *Berberis microphylla* secondary metabolites, root extract, berberine and palmatine (Manosalva et al., 2014), were dissolved in methanol to a final concentration of 10 ppm for alkaloids isolated and 100 ppm for root extract. Five grams of diet were mixed with 1 ml of different treatments in Petri dishes, equivalent to 0.0002% for berberine and palmatine and 0.002% for root extract and the solvent was allowed to evaporate for 6 h in dark. In the control experiment, the insects received the same quantity of methanol used as solvent for preparing the treatments. Then 40 third instar *G. mellonella* larvae were put into each Petri dish, and 15 larvae were chosen after 24 and 48H for the enzymatic extraction. These experiments were repeated three times.

### Acetylcholinesterase (AChE) activity

The effect on AChE by root extract and isolated compounds was determined using the spectrophotometric method according to Ellman et al. (1961) with modifications. Fifteen larvae heads were recollected and homogenized in a plastic pestle homogenizer in ice-cold extraction buffer (pH 7.5) containing 250 mm sucrose, 50 mm Tris-HCl, 1 mm EDTA, and 1 mm DTT at a 1:4 w/v ratio. The homogenates were centrifuged at 9,000 g for 25 min at 4°C. The supernatants were collected and stored at −80°C until analysis. Fifty microliters of supernatants were added to the wells. The plates were incubated for 30 min at 25°C before the addition of 100 μl of the substrate solution (0.04 M Na_2_HPO_4_, 0.2 mm, 5,5′-dithio-bis-2-nitrobenzoic acid (DTNB), 0.24 mm acethyltiocholine iodide (ATCI) in HPLC-grade water. After 5 min, the absorbance was read at 405 nm in a microplate reader (BIOBASE-EL10A). Results were expressed as units per minute per milligram proteins (UE/min mg proteins), where a unit of enzyme activity was defined as the change of 0.001 in the absorbance value under the assay conditions. The experiment was repeated three times.

### Carboxylesterase (CarE) activity

The CarE activity was measured according to Chen et al. (2017). Fifteen larvae from *G. mellonella* were recollected and homogenized with a plastic pestle homogenizer in ice-cold extraction phosphate buffer (0.04 M, pH 7.0). The homogenates were then centrifuged at 12,000 rpm for 30 min at 4°C. The supernatant was collected and diluted 50× with phosphate buffer for CarE assay. The enzyme reactions contained 50 μl of the diluted supernatant, 450 μl of 0.04 mol/L phosphate buffer, and 3.6 ml substrate (α-naphtyl acetate), which were mixed and incubated in a shaker for 30 min at 37°C. Then, 900 μl of chromogenic reagent (1% FastBlue B salt-SDS) was added, and the reactions were incubated for a further 30 min at room temperature, followed by measurement of absorbance at 600 nm. Known concentrations of α-naphthol (0.004–0.02 mm) were used to obtain a standard curve. Results were expressed as nmol α-naphthol produced per minute per milligram of proteins. The experiment was repeated three times.

### Glutathione transferase (GST) activity

The GST activity was measured according to Pan et al. (2016) with some modifications. Fifteen larvae from *G. mellonella* were recollected and homogenized in a plastic pestle homogenizer in ice-cold extraction buffer (pH 7.5) containing 250 mm sucrose, 50 mm Tris-HCl, 1 mm EDTA, and 1 mm DTT at a 1:4 w/v ratio, which was then centrifuged at 12,000 rpm for 30 min at 4°C. The supernatant was considered the enzyme solution. The enzyme reaction was measured in a cuvette containing 820 µl phosphate buffer (66 mmol/L, pH 7.0), 35 µl enzyme solution, 105 µl of 50 mmol/L glutathione (GSH), and 40 µl of 30 mmol/L 1-chloro-2,4-dinitrobenzene (CDNB), and changes in absorption at 340 nm were recorded for 5 min. The results were expressed as nanomoles GSH-CDNB produced per minute per milligram of proteins. The measurement was repeated three times.

### Cytochrome P450 (CYP) activity

Cytochrome P450 (CYP) activity was measured using the method described by Pan et al. (2016) with modifications. Protein content was measured according to Bradford’s method (Bradford, 1976) using bovine serum albumin as the standard. Fifteen larvae from *G. mellonella* were collected and homogenized in 500 μl ice-cold homogenization buffer (10% glycerol in 0.1 mol/L phosphate buffer, pH 7.5). Then, the homogenate was centrifuged at 15,000 rpm for 30 min at 4°C, and the supernatant was collected for enzyme activity measurements. 20 µl *p*-nitroanisole as a substrate for CYP was added to 100 µl of the supernatant. To this 50 µl of NADPH was added in the dark and the tubes were left to incubate for 30 min. The reaction was stopped by adding 500 µl of sodium hydroxide. The reaction mixture was centrifuged at 10,000 rpm for 30 min at 4°C. The absorbance of the supernatant was determined at 400 nm. The results were expressed as µg *p*-nitrophenol released per minute per milligram of proteins. The activity of each sample was measured three times.

### Identification and annotation of GSTs from transcriptomic data

To identify amino acid sequences for GSTs, three pieces of RNA-seq data were used, where two were obtained from NCBI database (https://www.ncbi.nlm.nih.gov/). An antennal transcriptome reported by Zhao et al. (2019) and retrieved from Bioproject PRJNA616199 (SRA study SRP254512) as well as larval transcriptome from 454 Sequencing data within the Bioproject PRJEB2452 (SRA study ERP000555) were used. Finally, an assembled adult head transcriptome from our laboratory and reported in Lizana et al. (2020) was used. Homology searches were performed using a manually built amino acid database in FASTA format for GSTs from moths, such as *B. mori*, *Helicoverpa assulta,* and *Amyelois transitella*. The database was prepared using local BLAST through makeblastdb script for proteins. Subsequently, transcripts were identified by local searches using the Tools of the NCBI BLASTx (Altschul et al., 1997) between our databases and the assembled data set from each *G. mellonella* transcriptome (i.e., antennae, head and larvae). BLAST hits with e-values <1.0E-5 were considered significant (Anderson and Brass, 1998), and transcripts were assigned to each contig based on the BLAST hits with the highest score value. The open reading frame (ORF) of each unigene was determined using the ORF finder tool (https://www.ncbi.nlm.nih.gov/orffinder). Once *GST*-related transcripts were identified for each assembled transcriptome, comparisons among sequences were performed using multiple sequence alignments through Clustal Omega (https://www.ebi.ac.uk/Tools/msa/clustalo/) and phylogeny through FastTree software (Price et al., 2010) to detect repeated sequences (Supplementary Table S2).

### Phylogenetic analysis

A phylogenetic tree was constructed with MEGA X for each identified GST (Kumar et al., 2018). Thus, GST-related amino acid sequences from moths, namely *B. mori*, *S. littoralis*, *S. litura*, *P. xylostella*, *Cydia pomonella*, and *Eriocrania semipurpurell*a (Durand et al., 2018; Hu et al., 2020) were aligned against *G. mellonella* GSTs using MAFFT server (https://mafft.cbrc.jp/alignment/server/) (Katoh et al., 2019). Phylogeny was tested using the maximum-likelihood method with a 1,000 bootstrap analysis along with the Jones-Taylor-Thornton (JTT) model (Jones et al., 1992). To highlight clades, specific taxa and classification, the phylogenetic tree was edited using the iTOOL server (https://itol.embl.de/).

### Relative expression by qRT-PCR

To test the relative expression of the identified *GSTs*, third instar *G. mellonella* larvae (*n* = 6) were treated with berberine for 48 h at a 0.0002% concentration (equivalent to 10 ppm in methanolic solution) added to the diet in three replicates. A group of larvae (*n* = 6) were kept without treatment as negative control. Total RNA extraction was performed with Trizol (Invitrogen, United States). From the extracted RNA, cDNA was synthesized using 1 μg of total RNA using a qPCR AffinityScript (Stratagene, United States) cDNA synthesis kit according to the manufacturer’s recommendations. For qRT-PCR, primers were designed using the Primer3 platform (http://bioinfo.ut.ee/primer3-0.4.0/) (Supplementary Table S3) and their efficiencies (ranging from 90 to 110%) were validated by a standard curve with five 5× serial dilutions of the antennal cDNA of *G. mellonella*. An AriaMx Real-time PCR system (Agilent, Santa Clara, United States) was used for all the reactions, which were prepared using the Brilliant II SYBR Green qPCR Master Mix (Agilent, Santa Clara, United States) based on the manufacturer’s protocol. Cycle conditions were as follows: 95°C for 10 min followed by 40 cycles at 95°C for 3 s, 60°C for 30 s and for the melting curve 95°C for 15 s, 60°C for 1 min and 95°C for 15 s. It is worth noting that the minimum requirements for qRT-PCR according to MIQE guidelines were followed (Bustin et al., 2009). Thus, the presence of only one amplified PCR product was verified for each reaction by melting curve (95°C for 15 s, 60°C for 1 min and 95°C for 15 s). A housekeeping gene, β-actin, was used as an internal control. All experiments were performed using three biological replicates, each with three technical replicates, and the relative quantification between treated and untreated larvae was analyzed using the 2^−ΔΔCt^ Pffafl method (Pfaffl, 2001).

### Homology modeling, refinement, and molecular docking

To study the three-dimensional (3D) arrangement of the selected GSTs and its active sites in depth, 3D models were built and used as a target to dock berberine according to the methodology reported by Venthur et al. (2019). Thus, protein models for *G. mellonella* GSTs were obtained using *D. melanogaster* GST (PDB code: 4PNG) as template through the Modeller v9.18 software (Webb & Sali, 2016). Thus, 500 models were retrieved and the structure ranked with the lowest Discrete Optimized Potential Energy (DOPE) score was selected for further molecular dynamics with Not (just) Another Molecular Dynamics (NAMD) v2.9 Software and Chemistry at Harvard Macromolecular Mechanics (CHARMM36) force fields (Phillips et al., 2005). Briefly, GST models were solvated with water (TIP3P model) in a cubic box with a distance of 20 Å between the protein and the edge of the box. The system net charge was neutralized by adding Na^+^ or Cl^−^ randomly placed in the box. Likewise, the system was simulated under periodic boundary conditions with a cut-off radius of 12 Å for non-bonded interactions and a time step of 2 fs. Alpha-carbons (Cα) of secondary structures were fixed with a constant force of 1 kcal/mol/Å. A first energy minimization of 50,000 steps was performed and followed by heating through short simulations of 1 ps at 50, 100, 150, 200, 250, and 300 K. Long simulations were kept at 300 K and 1 bar pressure in the NTP (referred to a constant number of particles, temperature and pressure) during 100 ns. The RMSD trajectory tool was used to estimate stability during simulations with reference to the starting structure, and when RMSD did not show any big changes, the coordinates were analyzed *via* ProCheck (Laskowski et al., 1993). Likewise, energy minimization for berberine was performed using MM2 minimization methods present in the Chem3D software (Perkin Elmer). For the GST model, polar hydrogens were added using the AutoDock Tools interface as well as torsional bonds for ligands. Two grid boxes with a default space of 1 Å were prepared via AutoGrid, one with 54 × 54 × 54 points that covers the entire protein structure (i.e., blind docking) and one with 28 × 28×28 points that delimits the binding site volume (refined docking). Docking runs were carried out using AutoDock Vina (Trott and Olson, 2010) with exhaustiveness set to 500. The best binding modes were selected according to the lowest free binding energy (kcal mol^−1^). To support molecular docking and identification of potential binding sites, the CASTp calculation server (http://sts.bioe.uic.edu/castp/calculation.html) was used.

### Statistical analysis

Statistical differences were evaluated by *t*-test using the SPSS Statistics 22 software (SPSS Inc, Chicago, IL, United States) in both enzymatic assays and qRT-PCR experiments. Likewise, a one-way ANOVA was implemented for enzymatic assays to compare treatments using the same software.

## Results

### Effect of alkaloids on larvae

Alkaloid extract from *B. microphylla* resulted in seven different components: isocorydine, berbamine, calafatine, protopine, jatrorrhizine, palmatine and berberine (Table 1). It is worth noting that only calafatine, jatrorrhizine, palmatine and berberine could be identified against standards, with the last two being available. Besides this, berberine has been reported as the most abundant isoquinoline alkaloid in stem and roots of *B. microphylla* (Manosalva et al., 2014). With this in mind, larvae of *G. mellonella* were treated with palmatine and berberine to test effects on mortality, pupation and diet ingestion. From both exposed and non-exposed larvae, observations suggest strong resistance to the alkaloids. No significant differences were found between exposed and non-exposed larvae in terms of mortality. A trend to early pupation was observed in the first eight recorded days along with 5% less diet consumption among the alkaloid-exposed larvae (Supplementary Figure S1; Supplementary Table S1). These observations led us to hypothesize a strong enzymatic activity to detoxify alkaloids. Therefore, further experiments were focused on the identification of detoxification enzymes responsible for alkaloid transformation.

**TABLE 1:** Mass Spectral data and retention times (tR) of alkaloids identified from roots of *B. microphylla* by HPLC ESI-MS/MS.

Compounds	tR (min)	[M + H]^+^	[M]^+^	*m/z* fragment ion (% base peak)
Aporphine-type				
Isocorydine^a^	13.08	342		190(54), 207(36), 248(18), 222(27), 264(27),265(72), 279(100), 296(36), 311(27)
Bisbenzylisoquinoline-type				
Berbamine^a^	15.5	609		381(23), 578(1), 609(100)
Calafatine^b^	16.6	653		610(62), 622(100)
Protopine-type				
Protopine^a^	14.2	354		149(3), 177(12), 189(100), 206(18),247(18), 275(37)
Protoberberine-type				
Jatrorrhizine^b^	18.4		338	280(54), 294(100), 307(72), 308 (36) 322(100)
Palmatine^b^	19.6		352	279(2), 294(18), 308(57), 322(47), 336(100)
Berberine^b^	20.0		336	205(1), 234(2), 263(9), 275(11), 278(62), 292(78), 320(100), 306(59)

tR = Retention time.^a^ MS, data of literature;^b^ authentic standard.

### Enzymatic activity for alkaloid detoxification

**FIGURE 1:**
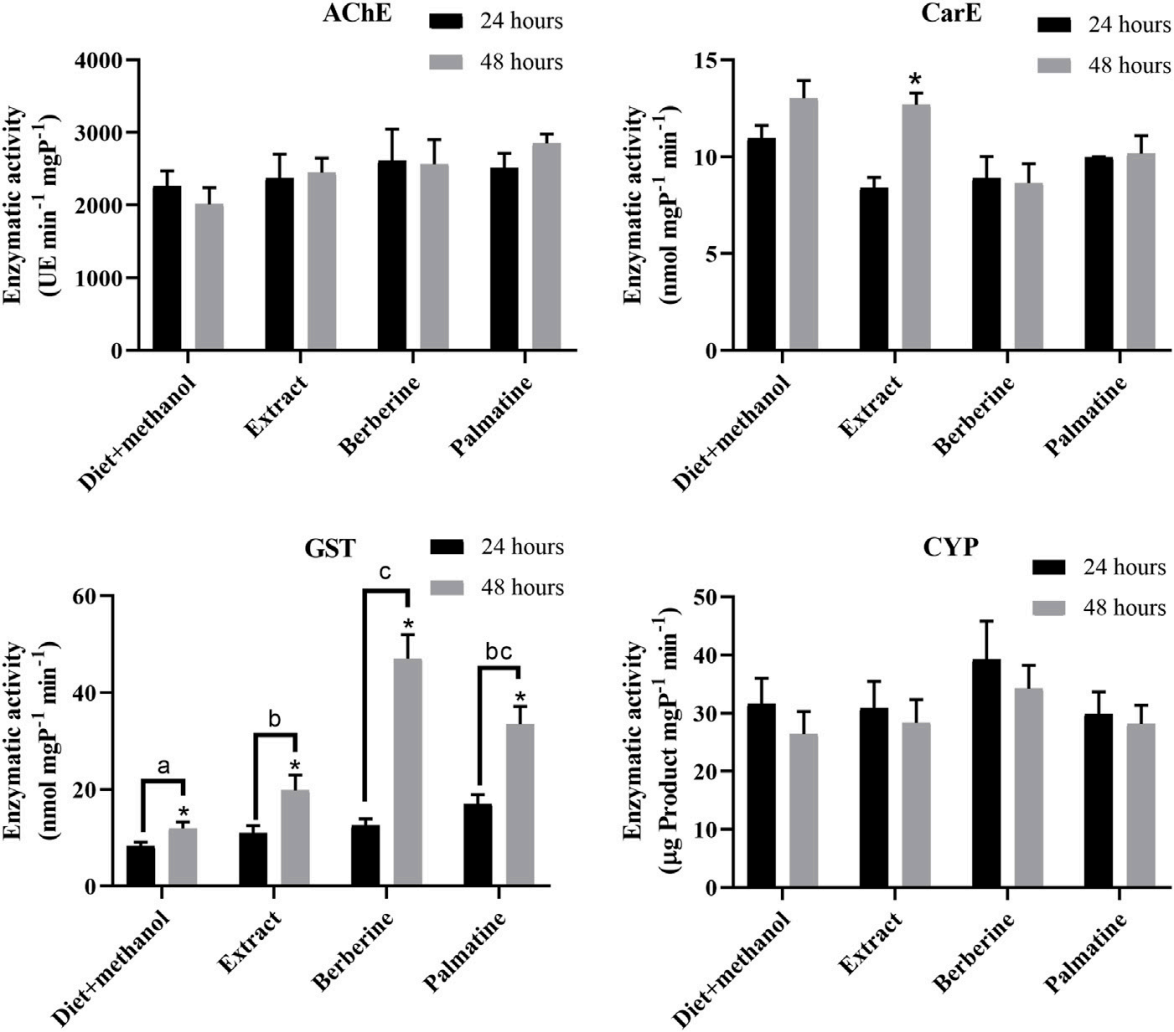
Enzymatic activity measured for carboxyl esterases, acetylcholinesterase, glutathione-S-transferase and cytochrome P450 during 24 and 48 h against *B. microphylla* extract, berberine, palmatine and diet + methanol (negative control). Different letters indicate statistically significant difference among treatments based on one-way ANOVA analysis. Asterisk (*) indicates statistically significant difference between 24 and 48 h for each treatment based on *t*-test analysis.

To identify highly active detoxification enzymes, enzymatic assays were performed where AChE, CarE, CYP, and GST activities were measured. Here, a root extract plus alkaloids berberine and palmatine from *B. microphylla* were used as a means to sort out enzymatic activity. Overall, no difference in activity was identified for AChE or CarE in terms of time (24 and 48 h) and substrate (extract or alkaloid), with the exception of CarE activity in the extract at 48 h (Figure 1). Similarly, CYP activity was measured for this study as such due to their involvement in xenobiotic catabolism (Scott, 1999; Shields et al., 2008). However, our findings suggest no difference in activity in terms of time or substrate (Figure 1). Finally, our enzymatic assays showed significant activity of GSTs at 48 h rather than 24 h, where berberine treatment resulted in greater enzymatic activity, especially at 48 h (Figure 1). In addition, the berberine treatment was significantly higher than either diet plus methanol or *B. microphylla* extract treatments. Although palmatine treatment appeared to have a statistically similar effect as berberine, no significant difference was found to *B. microphylla* extract treatment.

### Identification of G. mellonella GSTs and phylogenetic analysis

**FIGURE 2:**
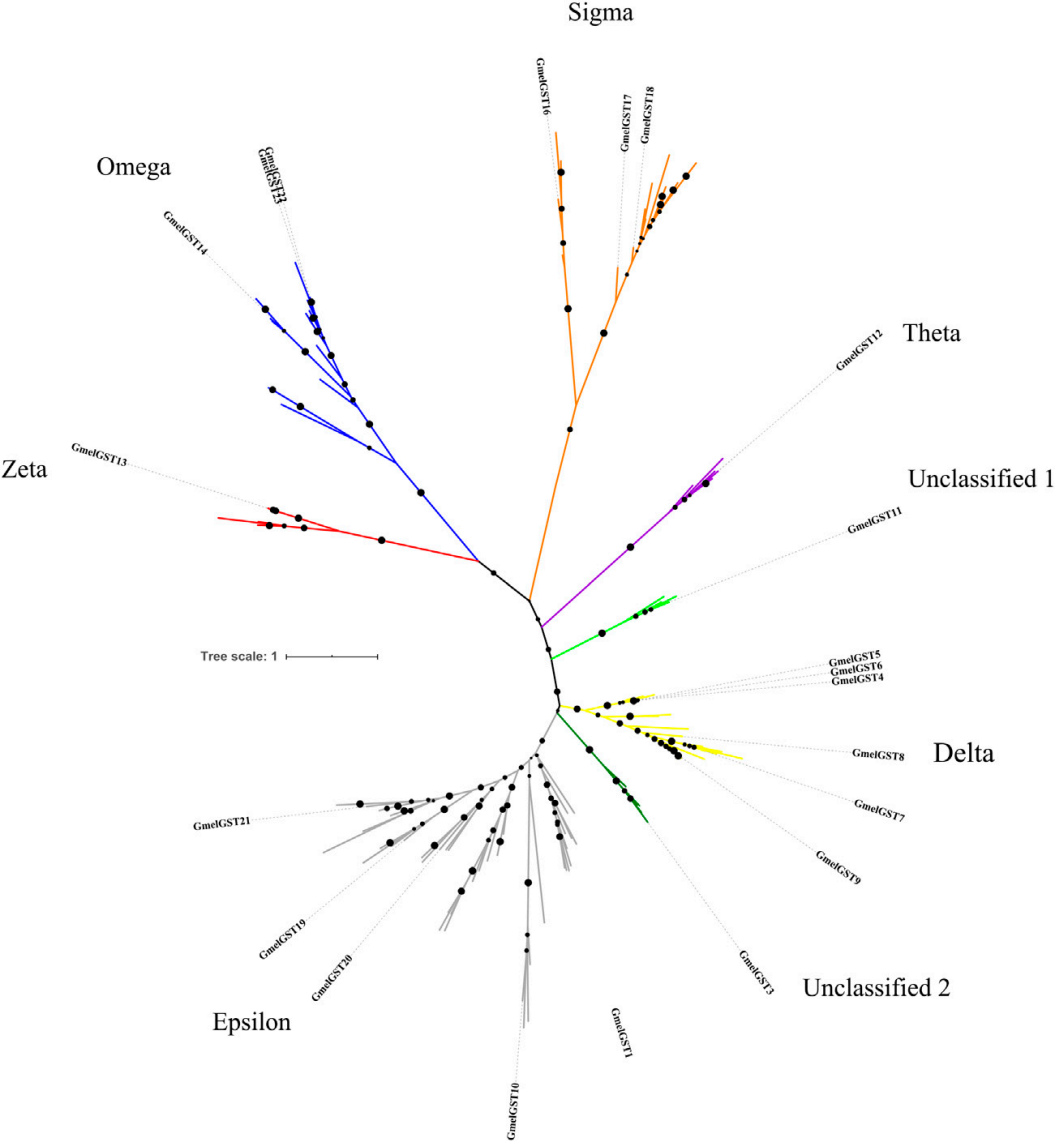
Phylogenetic tree for glutathione-S-transferases identified from *G. mellonella* transcriptomes. Different GST classes are indicated in red (zeta), blue (omega), orange (sigma), purple (theta), light green (unclassified 1), yellow (delta), green (unclassified 2) and grey (epsilon). GmelGSTs are highlighted as bold letters.

Through BLASTx analysis, sequence alignment and initial phylogenetic relationships, 22 *GST*-related transcripts with a range of 174–1,386 nucleotides (nt), were identified. From those, 32 transcripts had a full length ORF with an average of 1,200 bp. Phylogenetic analysis showed all the *G. mellonella GST* (*GmelGST*) transcripts clustered in eight main clades, divided into classes, namely zeta (z), omega (o), sigma (s), theta (t), unclassified 1 (u1), delta (d), unclassified 2 (u2) and epsilon (e) (Figure 2). The GmelGST13 appeared in zeta class, while three GmelGSTs (i.e., GmelGST14, 21 and 22) appeared in omega class. Three other *G. mellonella* GSTs, such as GmelGST15, 16, and 17 are related to sigma class. The GmelGST12 appeared in theta class, and GmelGST11 and 3 as unclassified 1 and 2, respectively. Six *G. mellonella* GSTs appeared related to delta class, namely GmelGST4, 5, 6, 7, 8, and 9. Finally, GmelGST1, 10, 19, 18 and 20 appeared related to epsilon class. It is worth noting that sigma, delta and epsilon clades appeared with the greatest number of expansions.

### Upregulation of GSTs after berberine treatment

**FIGURE 3:**
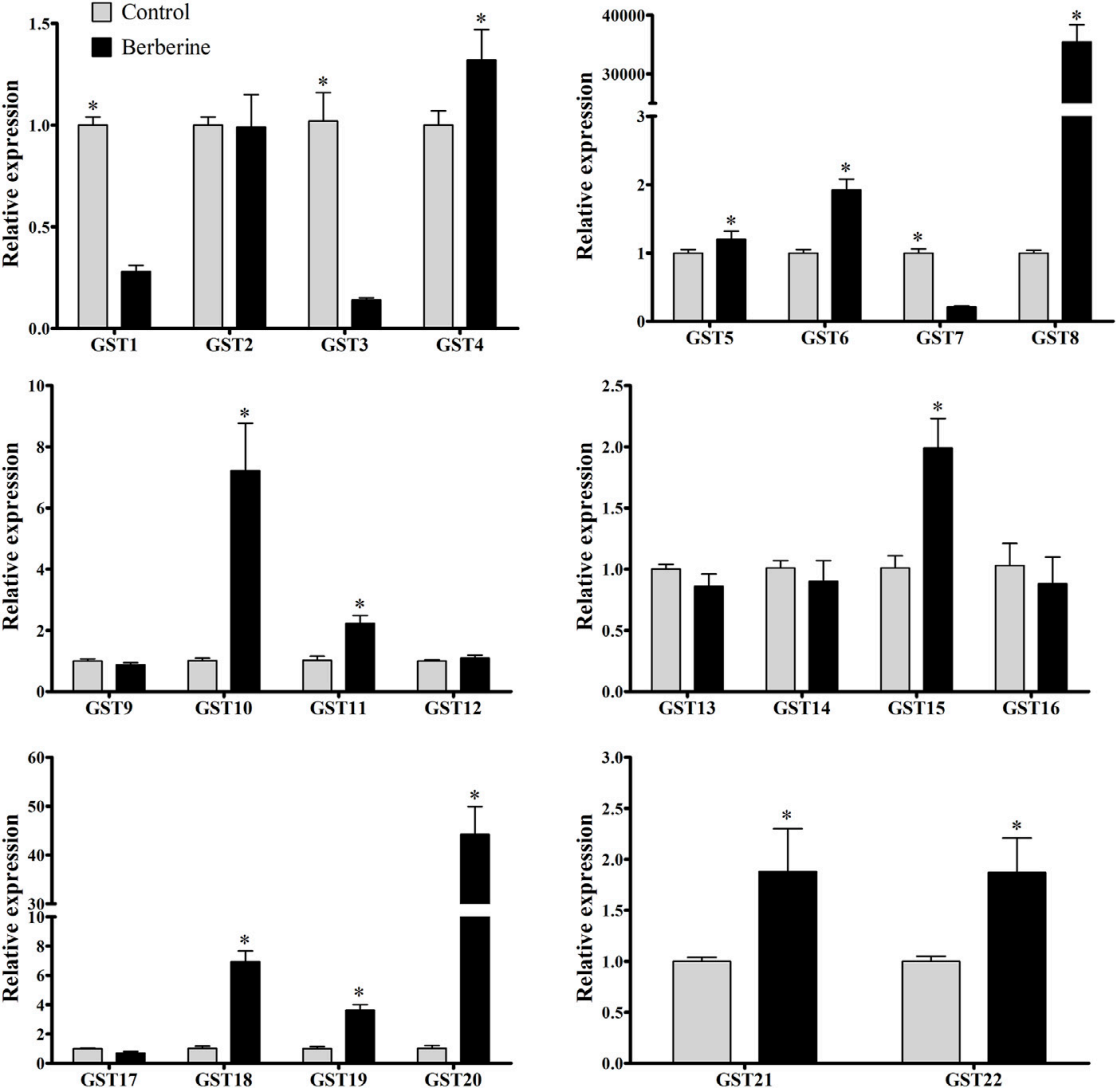
Relative expression of GSTs by qRT-PCR. Black and grey bars indicate larvae under berberine treatment and larvae under control treatment (water), respectively, based on the ratio of treatment to control. Statistically significant differences (*p* < 0.05) were evaluated by *t*-test and shown with asterisk (*) based on three biological replicates and three technical replicates.

Considering berberine is commercially available, highly abundant in roots of *B. microphylla*, and the alkaloid that showed the highest enzymatic activity for GSTs, it was selected for relative expression experiments. The expression of the 22 identified *GmelGSTs* showed three *GSTs* downregulated after berberine treatment (*GmelGST1*, three and seven). On the other hand, 12 *GmelGSTs* appeared upregulated, such as *GmelGST4*, *5*, *6*, *8*, *10*, *11*, *15*, *18*, *19*, *20*, *21*, and *22* (Figure 3). Remarkably, among the upregulated *GSTs*, *GmelGST8* and *20* showed the highest relative expression with a 35,400- and 44-fold increase, respectively. Other *GSTs*, such as *GmelGST10*, showed a 7-fold increase in expression. Likewise, a 1.25- to 7-fold increase in expression was obtained for *GmelGST4*, *5*, *6*, *11*, *15*, *18*, *19*, *21*, and *22*.

### 3D structure of GST8 and GST20

**FIGURE 4:**
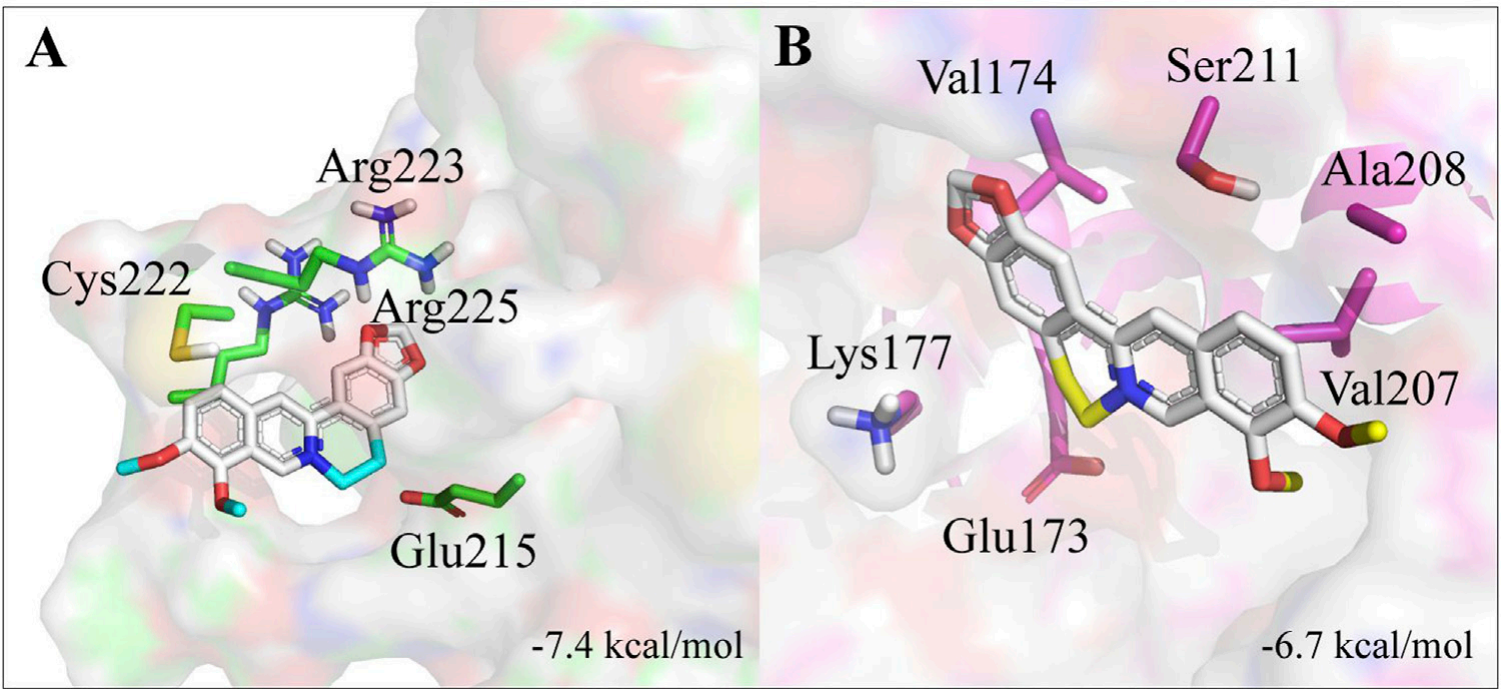
Homology models for GST8 and GST20. **(A)** 3D structure of GST8. **(B)** 3D structure of GST20. Residues from predicted active sites and close to berberine, are indicated.

The 3D structures for GmelGST8 and GmelGST20, as the most upregulated enzymes from relative expression experiments, were built using homology modeling. Here, a homology model was obtained from comparison with *Drosophila melanogaster* GST PDB code: 4PNG, which shares 38% sequence identity with the selected GmelGSTs. Overall, GmelGSTs presented a globular-like conformation with eight α-helices and four β-sheets for both GmelGST8 and GmelGST20. Docked conformations showed berberine bound to different binding sites in each GmelGST, where complexes berberine-GST8 and berberine-GST20 resulted in −7.4 and −6.7 kcal/mol of free binding energy, respectively (Figure 4). The closest residues to bound berberine were Glu215, Cys222, Arg223, and Arg225 in GST8, whereas Glu173, Val174, Lys177, Val207, Ala208, and Ser211 were the closest residues to bound berberine in GmelGST20.

## Discussion

The greater wax moth, *G. mellonella*, is a global beehive pest that has remained without either effective or environmentally friendly control methods. Recent advances have focused on the olfactory system of *G. mellonella* adults to understand its chemical ecology (Zhao et al., 2019; Lizana et al., 2020). However, less focus has been given to larval stages. Thus, in this study we address the potential control of *G. mellonella* larvae, this stage being the one that attacks beehives.

To follow an environmentally friendly control method, a root extract of *B. microphylla*, as a known source of alkaloids, such as berberine and palmatine, was used to evaluate its role as a control agent. Alkaloids have been reported to evoke aversive behavioral responses in insects. For instance, berberine has shown an antifeedant effect on gypsy moth larvae, *Lymantria dispar* (Shields et al., 2008). Such behavioral effects on *G. mellonella* larvae have not yet been evaluated using alkaloids, nor their physiological responses.

For *G. mellonella*, a powerful profile of gut enzymes has been suggested, considering that hundreds of chemical components are related to beeswax, such as esters, hydrocarbons, hydroxyl polyesters, alcohols and, more recently, long-chain fatty acids (Tulloch, 1980; Kong et al., 2019). Particularly, GSTs were reported to be upregulated in salivary glands of *G. mellonella* after polyethylene exposure, and likely acting as a response to stress conditions (Peydaei et al., 2020). However, their activity against alkaloids has not been tested so far. Thus, to measure the impact of *B. microphylla*-related agents, enzymatic activities were recorded. From the enzyme activities measured (i.e., CarE, CYP, AChE, and GST), GSTs showed the highest activity in response to berberine at both 48 h and compared with the other treatments. The role of GSTs against xenobiotic compounds has been reported for some moths. For example, recombinant *C. pomonella* GSTe3 (CpGSTe3) was able to metabolize insecticide λ-cyhalothrin (Hu et al., 2020). Likewise, the GSTE1 in *Spodoptera litura* (SlGSTE1) appears involved in the detoxification of phytochemicals, such as indole-3-carbinol and allyl-isothiocyanate as well as xanthotoxin, a furanocoumarin (Zou et al., 2016). Additionally, GSTs seem involved in semiochemical degradation. An example is *Grapholita molesta* GSTD1 (GmolGSTD1), which showed high degradation activity towards (*Z*)-8-dodecenyl alcohol, a pheromone component, and host plant volatiles like butyl hexanoate (Li et al., 2018). Although moth GSTs have been reported with notorious transformation activities against a wide range of chemical compounds, alkaloids have not been consistently tested against these types of enzymes.

It is interesting to note that *G. mellonella* larvae increase their enzymatic activity (i.e., CarE and GST) at 48 h of exposure to alkaloid extract, but only GSTs are significantly active against berberine and palmatine. This indicates that at least against alkaloids, GSTs could be responsible for degradation in *G. mellonella* larvae, probably by transforming alkaloids into ionic conjugated products usually less toxic than the original alkaloid. Taking this into account, our next question was which GmelGST has the alkaloid-metabolizing properties. In that sense, we performed bioinformatics analyses to identify and annotate *GST*-related transcripts from three sources of transcriptomic data. Furthermore, we performed phylogenetic analysis to clarify their GST-related classification. Thus, 22 GmelGSTs were identified and distributed in all classes (Figure 2). In particular, GmelGST3 appeared in the same clade as BmGSTu2, an enzyme reported in the detoxification of organophosphate insecticides (Yamamoto and Yamada, 2016). Likewise, GmelGST20 appeared closely related to SlGSTe1 in the e clade, previously mentioned as a detoxifying enzyme of host plant volatiles (Zou et al., 2016). Similarly, GmelGST18 was related to e clade along with CpomGSTe3, which was shown to metabolize insecticide λ-cyhalothrin (Hu et al., 2020). Furthermore, GmelGST7, eight and nine were part of d clade along with insecticide-detoxifying GSTs from *C. pomonella*, such as CpomGSTd1, CpomGSTd3 and CpomGSTd4 (Liu et al., 2014; Wang et al., 2019; Wei et al., 2020), whereas GmelGST4, 5 and 6, also part of d clade, were closely related to SlitGSTd2 and MsexGST-msolf1, both related to odorant-degrading functions (Rogers et al., 1999; Durand et al., 2018). Overall, the literature suggests that GSTs related to both d and e classes, functions as xenobiotic-detoxifying enzymes and odorant-degrading enzymes (Enayati et al., 2005; Durand et al., 2018). In addition to the most studied GST classes, d and e, other GmelGSTs appeared in z class (e.g., GmelGST13), the enzymes of which have been linked with phenylalanine and tyrosine catabolism (Board et al., 1997). On the other hand, GSTs in o class have been related to oxidative stress protection (Yamamoto et al., 2009), where GmelGST14, 21 and 22 were present. The GmelGST15, 16 and 17 appeared in s class that has been proposed to be active against toxic compounds and subsequent oxidative stress at the larval stage (Durand et al., 2018).

Considering the phylogenetic relationships found for GmelGSTs, it is interesting to note that d and e class (i.e., GST8 and GST20, respectively), appeared related to enzymes with a xenobiotic-detoxifying function. Although structural data are scarce, early research from DDT-metabolizing GST (agGSTd1-6) in mosquito *Anopheles gambiae* reported a fold similar to other insect d class GSTs (Chen et al., 2003), this being consistent with 57.77% of sequence identity between GST8 and agGSTd1-6. Our molecular modeling findings suggest that GST8 could be more predominant in berberine detoxification rather than GST20. However, alkaloid detoxification cannot be attributed to GST8 solely, and further functional experiments will be necessary to corroborate its activity.

Finally, the special combination of xenobiotic-resistant larvae, an alkaloid extract from *B. microphylla* and the role of *G. mellonella* as a beehive pest led us to identify such GSTs as alkaloid-metabolizing enzymes, expanding the function of d class GSTs from insecticides to alkaloids. We believe these findings represent a novel approach in which GST function can be disrupted, identifying and/or designing new analogs that can inhibit GSTs in the framework of integrated pest management strategies. In this sense, the present study has lain the foundation for future research.

## Data Availability

The datasets presented in this study can be found in online repositories. The names of the repository/repositories and accession number(s) can be found in the article/Supplementary Material.
