# Supplementary material for: Analysis of glutathione-S-transferases from larvae of Galleria mellonella (Lepidoptera, Pyralidae) with potential alkaloid detoxification function
Source: Front Physiol. 2022 Sep 6;13:989006. doi: 10.3389/fphys.2022.989006 (PMC9486002; doi:10.3389/fphys.2022.989006)
Supplement: Supplementary file 2 [file Table1.DOCX]

**Supplementary Information**

**Evidence of glutathione-S-transferases from larvae of *Galleria mellonella* (Lepidoptera, Pyralidae) with alkaloid detoxification function**

**Running title:** Alkaloid detoxification by GSTs in *G. mellonella*

Herbert Venthur^a,b^¶ Paula Lizana^c^ ¶, Loreto Manosalva^d^, Valentina Rojas^c^, Ricardo Godoy^c^, Adonis Rocha^e^, Iván Aguilera^e^, Rubén Palma-Millanao^f^, Ana Mutis^a,b,^*


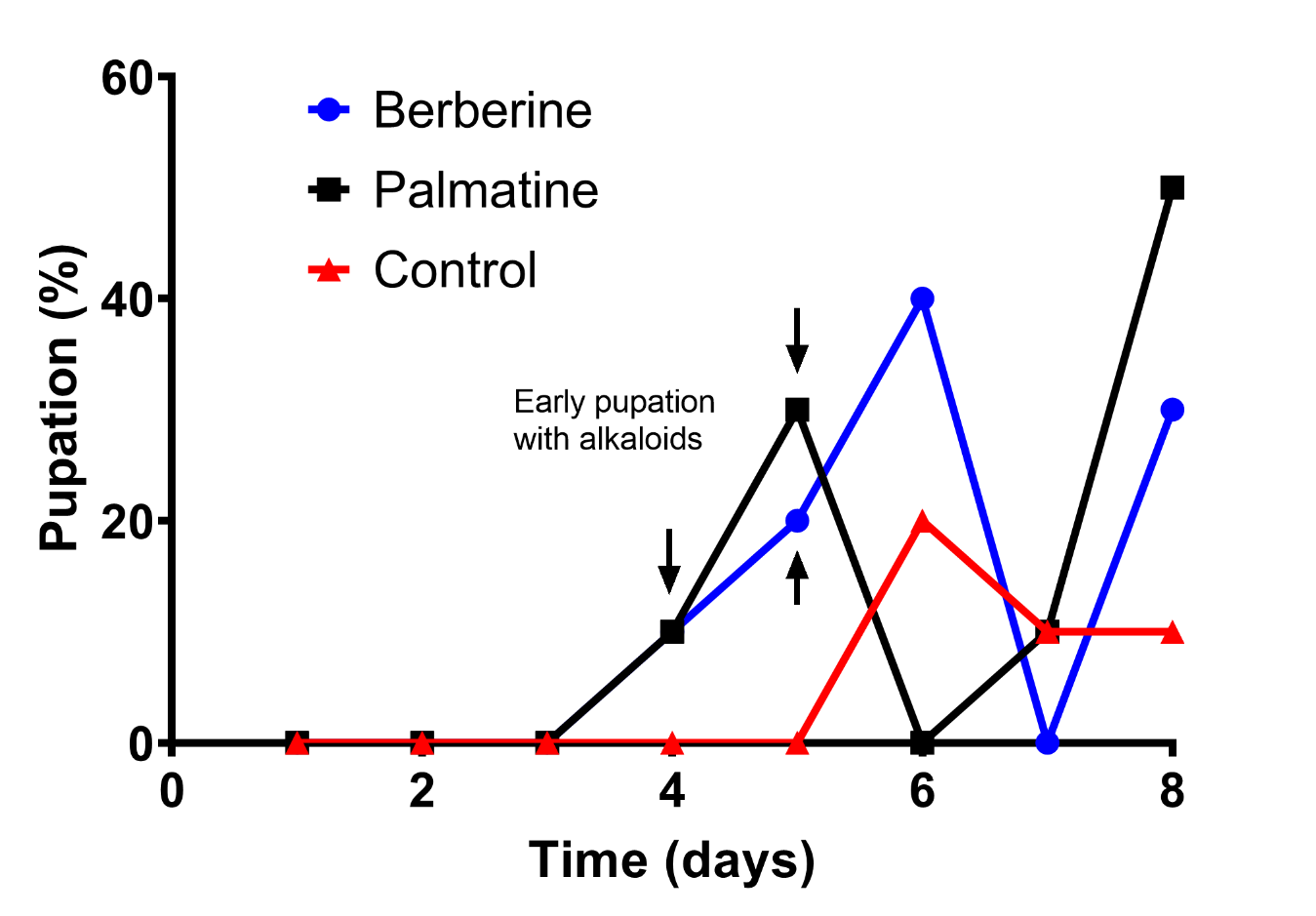


**Figure S1.** Larvae pupation recorded in 8 days upon exposition to berberine and palmatine.

**Table S1.** Diet consumption (%) of larvae of *G. mellonella* exposed to palmatine and berberine.

| Treatment (20 ppm) | Consumption (%) |
| --- | --- |
| Control^a^ | 38.2 ± 10.6 |
| Palmatine | 37.9 ± 6.6 |
| Berberine | 32.7 ± 8,0 |
| ^a^Negative control comprises water in diet instead of alkaloids. | |
